# The highest work rate associated with a predominantly aerobic contribution coincides with the highest work rate at which VO_2max_ can be attained

**DOI:** 10.1007/s00421-024-05533-z

**Published:** 2024-07-18

**Authors:** Arda Peker, Hakan As, Erkutay Kaya, Gorkem Aybars Balci, Ozgur Ozkaya

**Affiliations:** 1 Faculty of Sports Sciences, Ege University, 35050 Bornova, Izmir, Türkiye; 2 Institution of Health Sciences, Ege University, Bornova, Izmir, Türkiye; 3 AixTech Performance Lab, Ege University Technopark, Bornova, Izmir, Türkiye

**Keywords:** Anaerobic, Energy contribution, Extreme intensity, Severe, $\dot{\text{V}}$O_2_ ﻿kinetics

## Abstract

**Purpose:**

To estimate the highest power output at which predominant energy contribution is derived from the aerobic system (aerobic limit power: ALP) and to compare ALP with the upper boundary of the severe intensity exercise domain.

**Methods:**

Fifteen male individuals participated in this study. The upper boundary was estimated using *i)* linear relationship between time to achieve $\dot{\text{V}}$O_2max_ and time to task failure (P_UPPERBOUND_), *ii)* hyperbolic relationships between time to achieve $\dot{\text{V}}$O_2max_ vs. power output, and time to task failure vs. power output (P_UPPERBOUND_´), and *iii)* precalculated $\dot{\text{V}}$O_2max_ demand (I_HIGH_). ALP was estimated by aerobic, lactic, and phospholytic energy contributions using $\dot{\text{V}}$O_2_ response, blood [lactate] response, and fast component of recovery $\dot{\text{V}}$O_2_ kinetics, respectively.

**Results:**

ALP was determined as the highest power output providing predominant aerobic contribution; however, anaerobic pathways became the predominant energy source when ALP was exceeded by 5% (ALP + 5%) (from 46 to 52%; *p* = 0.003; ES:0.69). The $\dot{\text{V}}$O_2_ during exercise at ALP was not statistically different from $\dot{\text{V}}$O_2max_ (*p* > 0.05), but $\dot{\text{V}}$O_2max_ could not be attained at ALP + 5% (*p* < 0.01; ES:0.63). ALP was similar to P_UPPERBOUND_ and P_UPPERBOUND_´ (383 vs. 379 and 384 W; *p* > 0.05). There was a close agreement between ALP and P_UPPERBOUND_ (r: 0.99; Bias: − 3 W; SEE: 6 W; TE: 8 W; LoA: − 17 to 10 W) and P_UPPERBOUND_´ (r: 0.98; Bias: 1 W; SEE: 8 W; TE: 8 W; LoA: − 15 to 17 W). ALP, P_UPPERBOUND_, and P_UPPERBOUND_´ were greater than I_HIGH_ (339 ± 53 W; *p* < 0.001).

**Conclusion:**

ALP may provide a new perspective to intensity domain framework.

## Introduction

Severe intensity exercise is characterised by the attainment of maximal oxygen value ($\dot{\text{V}}$O_2max_) (Burnley and Jones 2007; Poole and Jones 2012; Poole et al. 2016). Thus, severe intensity exercise domain encompasses exercise intensities from maximal metabolic steady state, i.e., critical power (CP), to the highest power output at which $\dot{\text{V}}$O_2max_ can be achieved (Hill et al. 2002; Caputo and Denadai 2008; Raimundo et al. 2019). Within the severe intensity exercise domain, time to achieve $\dot{\text{V}}$O_2max_ is inversely related to exercise intensity (Margaria et al. 1965; Hill et al. 2002). In the extreme intensity exercise domain (intensities above the ‘upper boundary’ of the severe domain) task failure intervenes before $\dot{\text{V}}$O_2max_ can be attained (Hill et al. 2002). Intrinsically, this upper bound represents a critical threshold for eliciting a combination of central and peripheral training adaptations based on $\dot{\text{V}}$O_2_ kinetics, EMG responses, etc. (Turnes et al. 2016b, a, c; Lisbôa et al. 2019; Norouzi et al. 2022). Furthermore, high-intensity interval training performed at the upper bound is effective not only in enhancing endurance performance but also in improving sprint performance (Turnes et al. 2016c). This synergistic effect develops both aerobic power and anaerobic capacity, thereby contributing to comprehensive physiological adaptations (Norouzi et al. 2022).

**Fig. 1:**
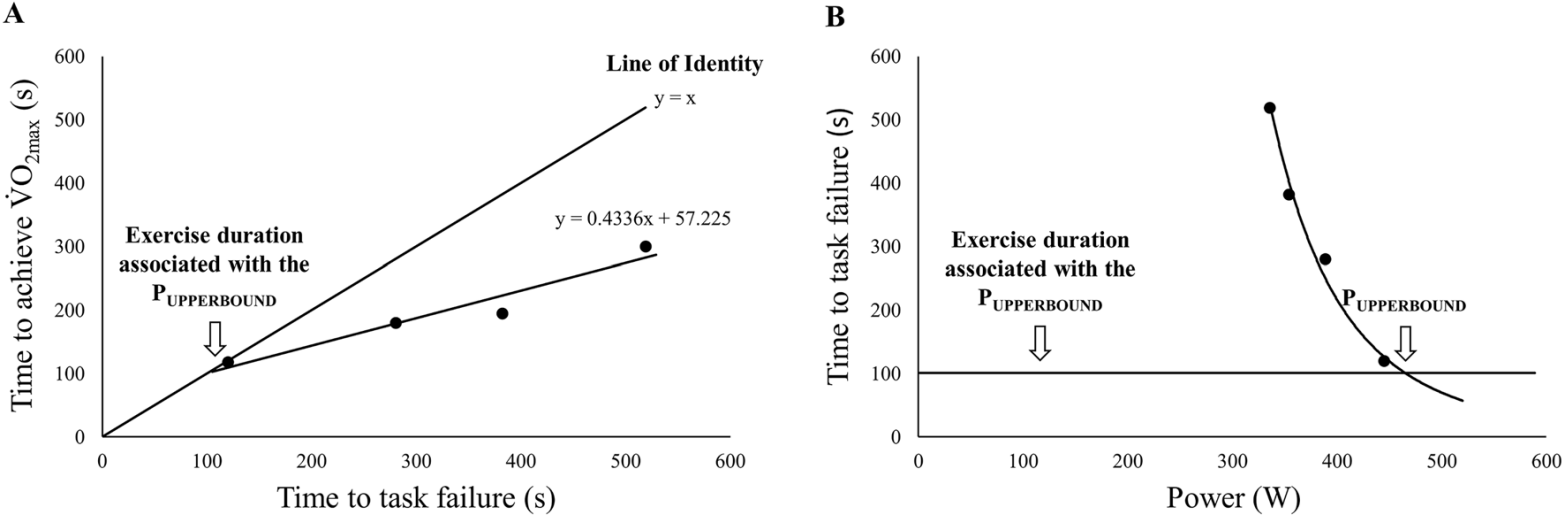
Estimation of the upper boundary of the severe exercise domain as proposed by Hill et al. (2002). In panel **A**, the point of the intersection between the time to achieve $\dot{\text{V}}$O_2max_ plotted against time to task failure and the line of identity demonstrates an exercise duration associated with the P_UPPERBOUND_. In panel **B**, the point of the intersection between the time to task failure plotted against power and estimated exercise duration is used to estimate the P_UPPERBOUND_. Data from a representative participant

**Fig. 2:**
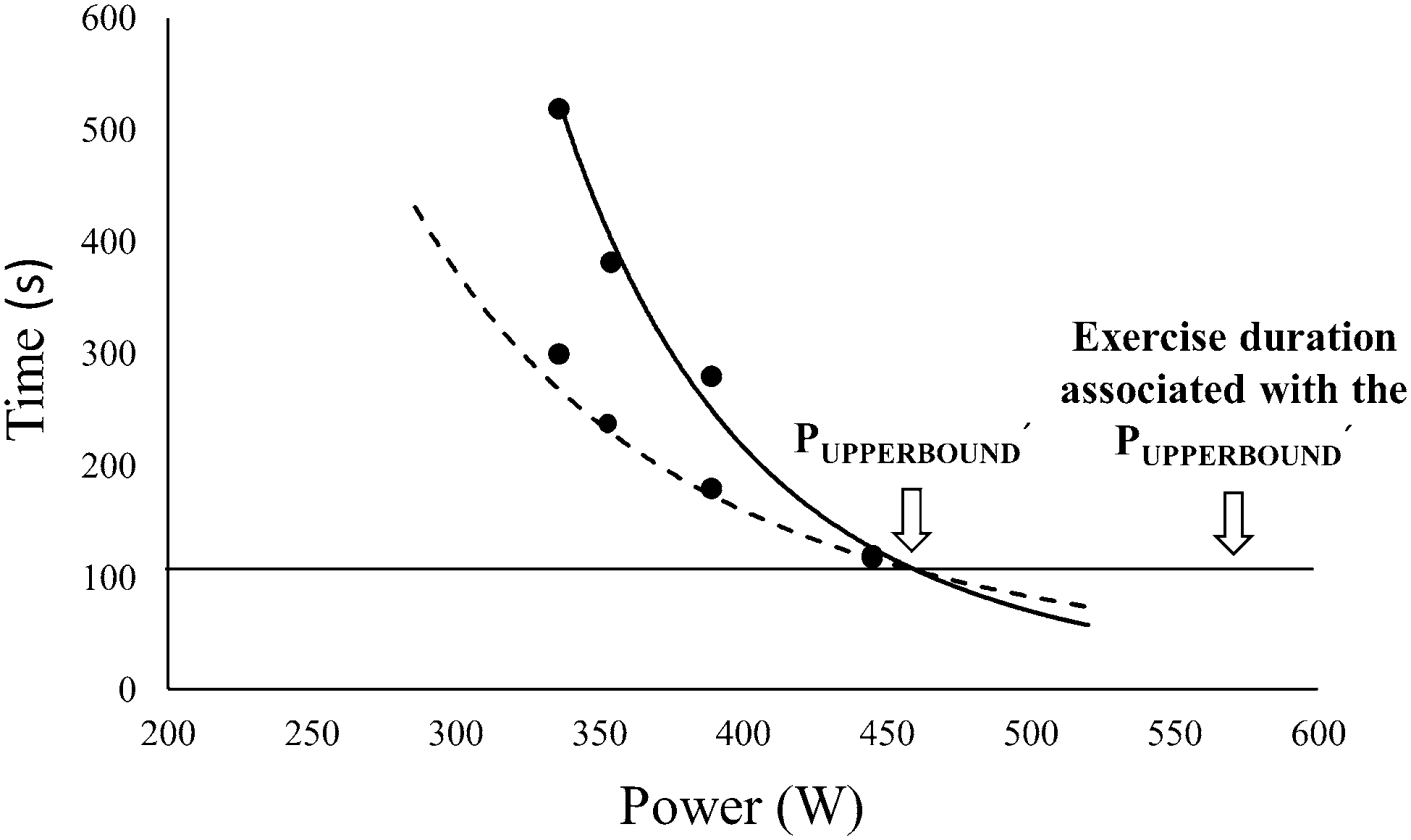
The upper boundary of the severe exercise domain as the intersection of the hyperbolic relationships between the time to achieve $\dot{\text{V}}$O_2max_ vs. power output (solid line) and time to task failure vs. power output (dashed line) as suggested by Hill and Ferguson (1999), Hill et al., (2002) and Hill et al., (2024). The projection of the intersection point of these two hyperbolic relationships provides an estimation for the time to task failure in y-axis and a power output in x-axis belonging to the upper boundary of the severe exercise domain (i.e., P_UPPERBOUND_´). Data from the same representative participant shown in Fig. 1

There are three essential approaches for assessing the upper boundary of the severe intensity exercise domain. One of these methods was proposed by Hill et al. (2002). In this method, the upper boundary is estimated by the relationship between the time to task failure (in x-axis) and time to achieve $\dot{\text{V}}$O_2max_ (in y-axis), obtained from 3–4 exhaustive exercise performed within the severe intensity exercise domain. In this relation, the intersection of the projected line with the line of identity (i.e., y = x) provides an estimation for the exercise duration of the highest power output at which $\dot{\text{V}}$O_2max_ can be attained momentarily (Fig. 1, panel A). Then, the external power associated with the upper boundary of the severe intensity exercise domain (i.e., P_UPPERBOUND_) is calculated by the point of the intersection between the hyperbolic power-time to task failure relationship and estimated time to task failure (Fig. 1, panel B). In addition, Hill and Ferguson (1999), Hill et al. (2002) and Hill et al., (2024) also reported that the upper boundary can be estimated by the intersection of the hyperbolic relationships between the time to achieve $\dot{\text{V}}$O_2max_ vs. power output, and time to task failure vs. power output (i.e., P_UPPERBOUND_´ in present study) (Fig. 2). The upper boundary is associated with an exercise duration of 100–160 s (Hill et al. 2002, 2024; Hill and Stevens 2005).

**Fig. 3:**
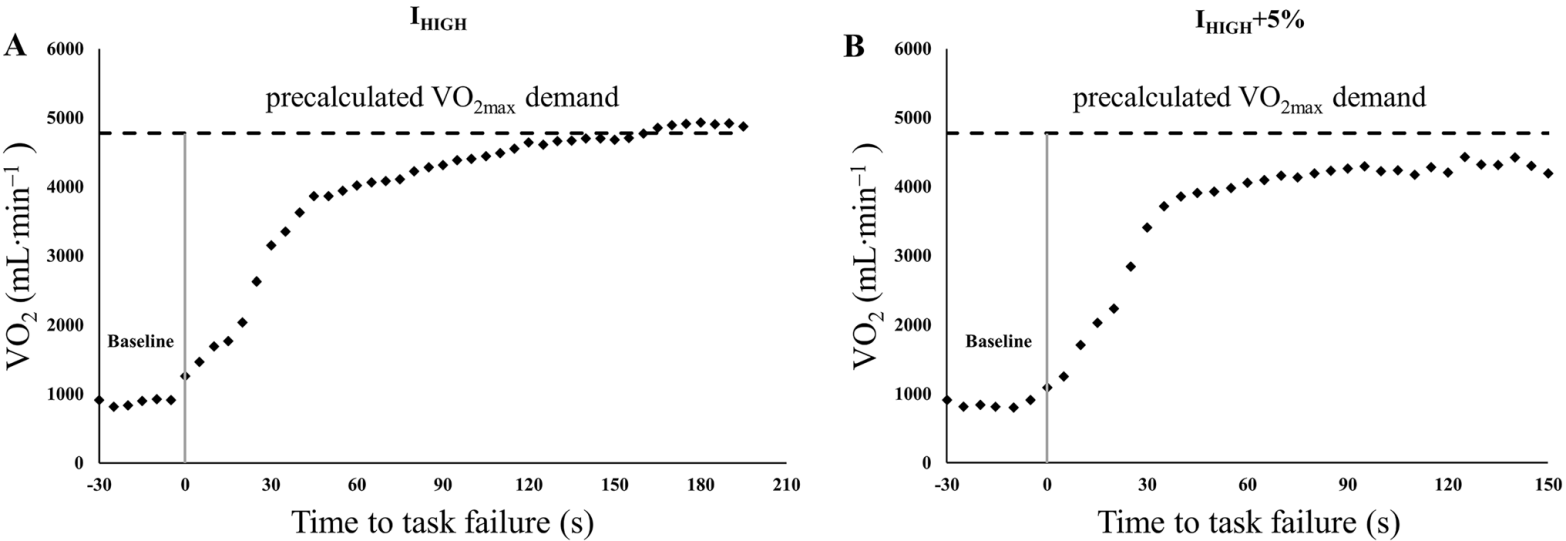
Estimation of the upper boundary of the severe exercise domain based on the method proposed by Caputo and Denadai (2008). The panel **A** indicates the highest exercise intensity at which precalculated $\dot{\text{V}}$O_2max_ demand could be achieved (i.e., I_HIGH_ as the upper boundary), and the panel **B** shows the lowest extreme-intense exercise at which precalculated $\dot{\text{V}}$O_2max_ demand could not be achieved (i.e., I_HIGH_ + 5% as a constant work rate exercise performed 5% above the I_HIGH_). Dashed line represents the precalculated $\dot{\text{V}}$O_2max_ demand calculated by subtracting one typical error of measurement from the average of the highest 15-s mean $\dot{\text{V}}$O_2_ values in panel **A** and **B**. Data from a representative participant

The third method for assessing the upper boundary of the severe intensity exercise domain was suggested by Caputo and Denadai (2008). In this method, the upper boundary is estimated by the data obtained from an exhaustive incremental exercise, and three further exhaustive exercise performed at constant work rates within the severe intensity exercise domain. Basically, the difference between the average of the highest $\dot{\text{V}}$O_2_ responses obtained from a total of four exercise and one typical error of measurement provides an attainable $\dot{\text{V}}$O_2max_ value within the severe intensity exercise domain. Then, the highest exercise intensity that elicits the $\dot{\text{V}}$O_2max_ is considered the upper boundary of the severe intensity exercise domain (i.e., I_HIGH_) (Fig. 3). Exercise duration at the I_HIGH_ was reported as 2–3.5 min (Caputo and Denadai 2008).

It is well-known that exercise resulting in task failure in less than ~ 120–130 s is supported by a predominant anaerobic contribution (Åstrand and Rodahl 1986; Serresse et al. 1988; Medbø and Tabata 1989; Bangsbo et al. 1990; Withers et al. 1991, 1993). It has also been shown that P_UPPERBOUND_, the highest work rate associated with the attainment of $\dot{\text{V}}$O_2max_, is associated with a tolerable exercise duration of 100 to 160 s (Hill et al. 2002, 2024; Burnley and Jones 2007; Poole and Jones 2012; Ozkaya et al. 2023). Thus, we hypothesised that the upper boundary of the severe intensity exercise domain, i.e., the upper end of the aerobic exercise zone, may be considered the highest exercise intensity at which predominant, i.e., 50%+, energy contribution is derived from the aerobic energy system (aerobic limit power: ALP), and ALP may denote a transition from the severe to extreme intensity exercise domain. If so, it should be noticed that exercise performed just above the ALP may provide a more appropriate exercise stimulus to enhance anaerobic capacity rather than improving aerobic power due to 50%+ anaerobic energy contribution and insufficient time spent at $\dot{\text{V}}$O_2max_. However, to date, ALP has yet to be compared with previous methods that are typically used to assess the upper boundary of the severe intensity exercise domain. Consequently, the aim of this study was to estimate the ALP and compare it with the P_UPPERBOUND_, P_UPPERBOUND_´ and I_HIGH_ to understand whether the ALP could be a boundary that partitions severe from the extreme intensity exercise domain.

## Materials and methods

## Ethical approval

This study was approved by university ethics committee (19-12 T/60). Experimental procedures were designed according to the rules and principles of the Helsinki Declaration. After explaining the study’s procedures, risks, and benefits to all individuals, written informed consent was received from each participant using the approved guidelines and documentations.

## Participants

**Fig. 4:**
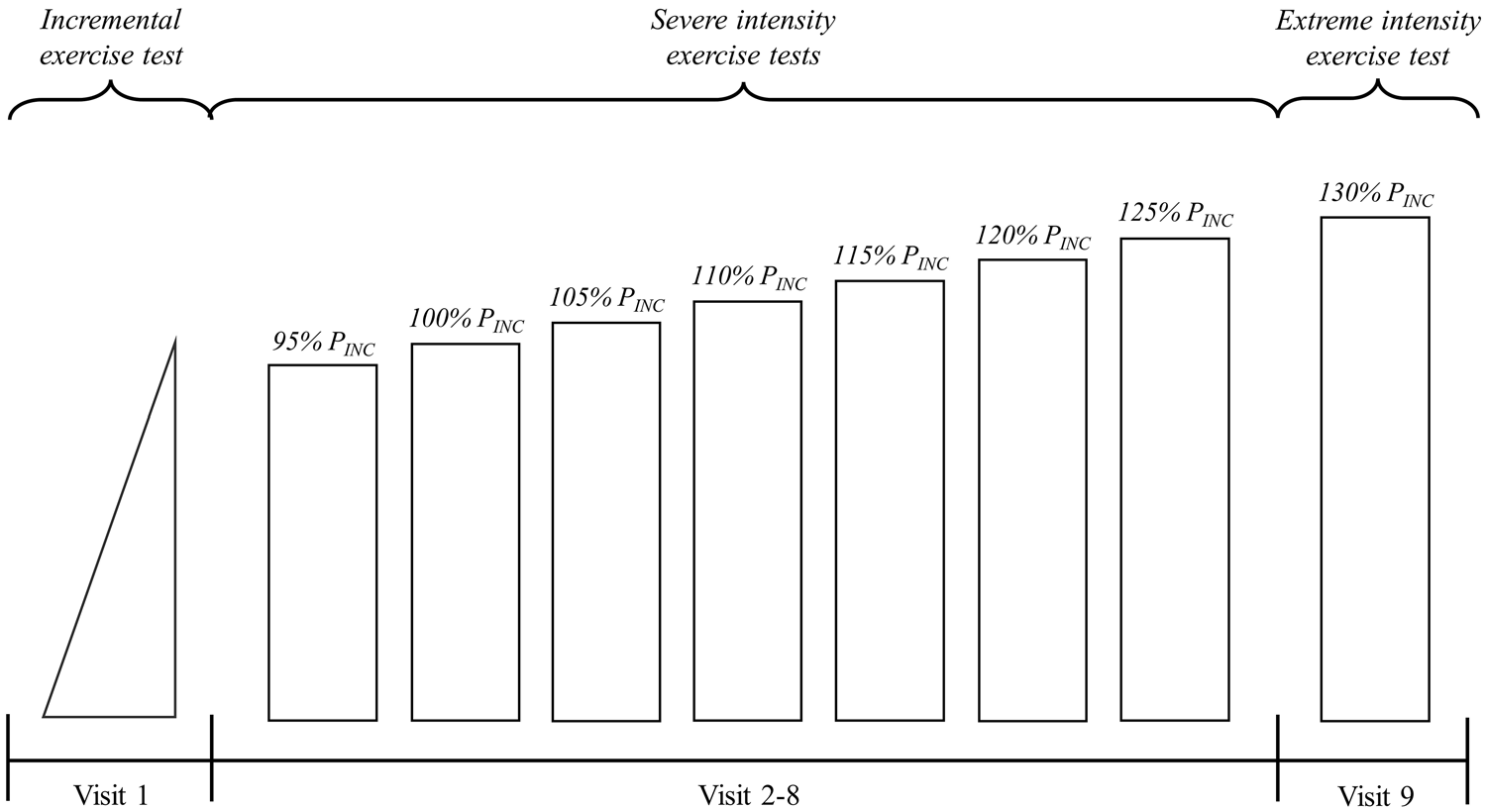
Schematic illustration of the experimental design. Over a period of 6–8-wk, each participant completed approximately nine excursions to the laboratory. These visits were separated by a 24–72-h rests. Following incremental exercise test session, each participant performed a series of constant work rate exercise tests until anaerobic energy metabolism became a major contributor. In this example, it seems that workload corresponding to 130% of P_INC_ provides a 50%+ anaerobic contribution (i.e., it is performed within the extreme exercise intensity domain). P_INC_: power output corresponding the highest 15-s $\dot{\text{V}}$O_2_ response at the end of the incremental exercise, calculated using the equation proposed by Faina et al. (1997) (qq. Eq. 1)

Fifteen physically active male individuals participated in this study (age: 31 ± 9 years; height: 1.77 ± 0.06 m; body mass: 70 ± 6 kg). They were required to visit the laboratory on 9–10 occasions over a 6- to 8-wk period with each visit separated by 24–72 h (Fig. 4). To ensure that circadian rhythm variance did not affect the results, the testing time was standardised (± 2 h) for each volunteer (Hill et al. 1992; Hill 2014). Over the course of the data collection, the participants did not alter their regular diet, sleep, or exercise habits. They were also instructed to refrain from any exhaustive exercise and to avoid drinking beverages containing alcohol or caffeine for 24 h before all trials. All participants had no history of systemic disease or injury and were not taking any medication during the study period.

## Data collection

The tests were performed on an electromagnetically braked cycle ergometer (Lode Excalibur Sport, Groningen, the Netherlands). Pulmonary gas exchange data were measured by breath-by-breath (Cosmed Quark CPET, Rome, Italy). Before each exercise testing session, the gas analyser was calibrated using ambient air and gas mixture of known concentration (16% O_2_, 5% CO_2_, and balanced N_2_). The turbine flow meter was calibrated with a 3-L calibration syringe. The breath-by-breath data were filtered to remove occasional errant breaths caused by swallowing, coughing, and sighing. After removing data points positioned more than four standard deviations (SD) from local mean (i.e., 5-breath rolling mean) (Lamarra et al. 1987), the data were smoothed using a 5-breath moving average (Hill 2019). Capillary blood samples (20 μl) were collected from the finger prick and immediately analysed by an automated blood [lactate] analyser (Biosen C-line, EKF Diagnostics, GmbH, Barleben, Germany). Samples were taken at baseline and immediately after the termination of constant work rate exercise bouts, as well as at the first, third, fifth, and seventh min of recovery period. Resting $\dot{\text{V}}$O_2_ responses were evaluated for 10 min in a seated position before each constant work rate trial, while post-exercise $\dot{\text{V}}$O_2_ responses were taken throughout 30-min seated position just following the termination of exercise. All exercise tests were conducted in a well-ventilated laboratory, under standard conditions (~ 20 °C temperature, ~ 20.8% O_2_, ~ 0.05% CO_2_, and 50–60% relative humidity).

## Procedures

### *Incremental tests*

On their first visit to the laboratory, participants performed a multi-stage incremental exercise test. The tests commenced with 4 min of baseline cycling without resistance. Then, 0.5 W·kg^−1^ workload was applied to the system and increased every 3 min by 0.5 W·kg^−1^. The incremental test was terminated when participants allowed the cadence to fall below 70 rpm for more than 10 s, despite verbal encouragement. Power output corresponding the highest $\dot{\text{V}}$O_2_ response (P_INC_) was calculated using the equation proposed by Faina et al. (1997) (Eq. 1).

1 $$ {\text{P}}_{\text{INC}}=\text{P}+\Delta \text{P}\times \left(\frac{\text{n}}{180}\right)$$

where P is the power output of the last step completed; ΔP is the increment of the power; and n is the number of seconds completed in the final stage.

### *Constant work rate exercise*

Constant work rate exercise tests were initiated at 95% of P_INC_ and the work rate was increased by 5% until anaerobic energy metabolism became a major contributor (50%+) on different days (qq. Fig. 4). Similar to the incremental exercise, the exercise tests commenced with 4 min of baseline cycling without resistance, then, workload was applied to the system. Each participant was encouraged to give maximum effort until the task failure. Exercise tests were terminated when participants allowed the cadence to fall below 70 rpm for more than 10 s. The highest 15-s $\dot{\text{V}}$O_2_ was recorded during tests.

### *Estimation of P*_*UPPERBOUND*_

Data obtained from constant work rate exercise tests terminated between 2 and 10 min (i.e., approximately performed at 95%, 100%, 110%, and 125% of P_INC_) were used for the estimation of P_UPPERBOUND_. Time to achieve $\dot{\text{V}}$O_2max_ was evaluated by nonlinear regression analysis (Sigma-Plot 14.0, Systat Software Inc., San Jose, CA, USA) using breath-by-breath $\dot{\text{V}}$O_2_ responses for each test (Eq. 2) (Hill et al. 2002; Hill and Stevens 2005).

2 $$\dot{\text{V}}O_{2\text{(t)}}=\dot{\text{V}}O_{2{\text{baseline}}}+{A}_{p}\bullet (1-{\text{e}}^{-(\text{t}-{\text{TD}}_{\text{p}})/{\uptau }_{\text{p}}})$$

where $\dot{\text{V}}$O_2(t)_ is the $\dot{\text{V}}$O_2_ responses achievable at any time during exercise; $\dot{\text{V}}$O_2baseline_ is the $\dot{\text{V}}$O_2_ obtained from the end of the baseline cycling period; A_p_ is the asymptotic amplitude of the primary phase which is the projected gain in $\dot{\text{V}}$O_2_; TD_p_ is the time delay during primary phase; and τ_p_ represents the time constant which is related to 63% of the final amplitude in $\dot{\text{V}}$O_2_ responses.

In order to calculate the time to achieve $\dot{\text{V}}$O_2max_, data belonging to the first 20 s of exercise (i.e., cardio-dynamic phase) were excluded from the analyses (Krogh and Lindhard 1913; Weissman et al. 1982; Ozyener et al. 2001). The point at which the amplitude of the mono-exponential curve reached 99% was considered to represent the maximum value of the $\dot{\text{V}}$O_2_ response following the primary phase (Hill et al. 2002). Time to achieve $\dot{\text{V}}$O_2max_ was evaluated as a value of 4.6 × τ. The point where the linear relationship (y = ax + b) between the time to achieve $\dot{\text{V}}$O_2max_ and time to task failure intersects with the line of identity (y = x, i.e., time to achieve $\dot{\text{V}}$O_2max_ = time to task failure) was accepted as an estimation for time to task failure (x = b × (1 − a)^−1^) of the upper boundary of the severe intensity exercise domain. For example, as shown in Fig. 1; x = 57.225 × (1 − 0.4336)^−1^ provided a time to task failure associated with P_UPPERBOUND_, i.e., 101 s for participant #9. Then, the point at which the hyperbolic relationship between the work rate and time to task failure intersects with the estimated exercise duration (i.e., 101 s) was considered the P_UPPERBOUND_, i.e., 458 W for participant #9 (Fig. 1) (Hill et al. 2002). On the other hand, as suggested by Hill and Ferguson (1999), Hill et al. (2002) and Hill et al. (2024), the upper boundary was also estimated as the point of the intersection of the hyperbolic relationships between the time to achieve $\dot{\text{V}}$O_2max_ vs. power output (i.e., P_UPPERBOUND_ = curvature constant × time to task failure^–1^ + CP) and time to task failure vs. power output (i.e., time to task failure = curvature constant × (P_UPPERBOUND_-CP)^–1^) (Fig. 2). Then, the projection of the intersection point of these two hyperbolic relationships provides an estimation for the time to task failure in y-axis and a power output (i.e., P_UPPERBOUND_´) in x-axis belonging to the upper boundary of the severe intensity exercise domain. For example, as shown in Fig. 2, P_UPPERBOUND_´ referred to 461 W and 104-s task failure for participant #9.

### *Estimation of I*_*HIGH*_

Data obtained from incremental exercise and further constant work rate exercise tests performed at 95%, 100% and 110% of P_INC_ were typically considered for the estimation of I_HIGH_ (Caputo and Denadai 2008). $\dot{\text{V}}$O_2max_ was calculated by subtracting one typical error of measurement from the average of the highest 15-s mean $\dot{\text{V}}$O_2_ values (Eq. 3). In order to calculate the typical error of measurement, the standard deviation of the highest 15-s mean $\dot{\text{V}}$O_2_ values obtained from incremental and constant work rate tests was divided by $\sqrt{2}$ (Caputo and Denadai 2008).

3 $$\dot{\text{V}}\text{O}_\text{2max}=\dot{\text{V}}\text{O}_\text{2avg}-\left(\frac{\dot{\text{V}}\text{O}_\text{2sd}}{\sqrt{2}}\right)$$

where $\dot{\text{V}}$O_2max_ is the difference between the average of the highest $\dot{\text{V}}$O_2_ values and one typical error of measurement; $\dot{\text{V}}$O_2avg_ is an average of the highest 15-s $\dot{\text{V}}$O_2_ values of incremental exercise and constant work rate exercise tests performed at 95%, 100% and 110% of P_INC_; and $\dot{\text{V}}$O_2sd_ is the standard deviation value of the mean.

Ultimately, participants implemented 2–4 further constant work rate trials until $\dot{\text{V}}$O_2max_ could not be attained or maintained. The I_HIGH_ was approved as the highest work rate and the shortest exercise duration providing the $\dot{\text{V}}$O_2max_.

### *Estimation of ALP*

The ALP was estimated as the highest exercise intensity at which predominant energy contribution is derived from the aerobic energy system. The net energy contribution rates from aerobic, lactic, and phospholytic pathways were evaluated to estimate the ALP. The net energy contribution of the aerobic energy pathway (W_AER_) was evaluated by the difference between integrated exercise and resting $\dot{\text{V}}$O_2_ data over time (Eq. 4) (Åstrand 1981; Beneke et al. 2002; Bertuzzi et al. 2007; Ozkaya et al. 2014).

4 $$ {\text{W}}_{\text{AER}}={\int }_{t0}^{t1}\dot{\text{V}}\text{O}_{2}-\dot{\text{V}}\text{O}_\text{2resting}$$

where t_0_ and t_1_ represent the time interval from the start to the end of the exercise; $\dot{\text{V}}$O_2_ is the total $\dot{\text{V}}$O_2_ responses during constant work rate exercise; $\dot{\text{V}}$O_2resting_ is the $\dot{\text{V}}$O_2_ response during resting condition; and W_AER_ is the net energy contribution of the aerobic energy pathway.

The net energy contribution of the phospholytic energy pathway (W_PC_) was assessed by the fast component of post-exercise $\dot{\text{V}}$O_2_ responses (Knuttgen 1970; Roberts and Morton 1978; Beneke et al. 2002; Bertuzzi et al. 2007; Ozkaya et al. 2014; Hill 2023). Post-exercise $\dot{\text{V}}$O_2_ response profile was evaluated using bi-exponential decay model (Eq. 5). The W_PC_ was then calculated using the area under the curve of the fast component of post-exercise the $\dot{\text{V}}$O_2_ response (Eq. 6).

5 $$ {\dot{\text{VO}}}_\text{2(t)}={\dot{\text{VO}}}_{2{\text{baseline}}}+{A}_{fc}\bullet {\text{e}}^{-(\text{t}-{\text{TD}}_{\text{fc}})/{\uptau }_{\text{fc}}}+{A}_{sc}\bullet {\text{e}}^{-(\text{t}-{\text{TD}}_{\text{sc}})/{\uptau }_{\text{sc}}}$$

where $\dot{\text{V}}$O_2(t)_ is the $\dot{\text{V}}$O_2_ responses achievable at any time during exercise; $\dot{\text{V}}$O_2baseline_ is the $\dot{\text{V}}$O_2_ at baseline; A_fc_ is the amplitude of the fast component of post-exercise $\dot{\text{V}}$O_2_ responses; τ_fc_ is the time constant of fast component of post-exercise $\dot{\text{V}}$O_2_ responses; TD_fc_ is the time delay during the fast component; A_sc_ is the amplitude of the slow component of post-exercise $\dot{\text{V}}$O_2_ responses; τ_sc_ is the time constant of slow component of post-exercise $\dot{\text{V}}$O_2_ responses; and TD_sc_ is the time delay during the slow component.

6 $$ {\text{W}}_{\text{PC}}={\int }_{\text{t}0}^{\text{t}1}{\text{A}}_{\text{fc}}\bullet {\text{e}}^{-(\text{t}-{\text{TD}}_{\text{fc}})/{\uptau }_{\text{fc}}}$$

where t_0_ and t_1_ represent the time interval from the peak $\dot{\text{V}}$O_2_ response of post-exercise recovery period to the end of the fast component; A_fc_ is the amplitude of the fast component of post-exercise $\dot{\text{V}}$O_2_ responses; τ_fc_ is the time constant of the fast component of post-exercise $\dot{\text{V}}$O_2_ responses; TD_fc_ is the time delay during the fast component; and W_PC_ is the net energy contribution of the phospholytic energy pathway.

The net energy contribution of the lactic energy pathway (W_LA_) was calculated by multiplying body mass, peak delta (Δ) [lactate], O_2_-[lactate] equivalent, and the caloric equivalent of O_2_ (di Prampero 1981; Mader and Heck 1986; Beneke and Meyer 1997; Beneke et al. 2002; Bertuzzi et al. 2007; Ozkaya et al. 2014; Hill 2023). Peak Δ[lactate] was considered the difference between exercise and resting status. The accumulation of 1 mmol·L^−1^ of blood [lactate] was accepted as the equivalent to 3 mL·kg^−1^·mmol^−1^·L^−1^ (di Prampero 1981; di Prampero and Ferretti 1999). The contribution of each energy system was expressed in terms of mL·kg^−1^ and kJ, assuming the caloric quotient of 21.1 kJ·L^−1^. Total energy expenditure during constant work rate exercise (W_Total_) was calculated as the sum of the energy derived from W_AER_, W_PC_, and W_LA_. Relative contributions of the energy systems were calculated as dividing the each of the W_AER_, W_PC_, and W_LA_ value by the W_Total_ (W_AER_%, W_PC_%, and W_LA_%, respectively). Absolute and relative total anaerobic energy contributions (W_ANE_ and W_ANE_%) were calculated as the sum of phospholytic and lactic contribution values. Note that the net energy contributions belonging to the ALP and I_HIGH_ were directly calculated during constant work rate exercise tests; however, the net energy contributions of each energy system for the P_UPPERBOUND_ and P_UPPERBOUND_´ were estimated by interpolated data obtained from the work rates just below and above the P_UPPERBOUND_ or P_UPPERBOUND_´ (i.e., within the range of 5%), since the P_UPPERBOUND_ and P_UPPERBOUND_´ was mathematically estimated.

## Statistical analyses

Variables were evaluated using SPSS 19.0 (SPSS Inc., Chicago, USA). Shapiro–Wilk test was used to examine whether the data were normally distributed or not. A one-way repeated measures analysis of variance was used to assess the differences between $\dot{\text{V}}$O_2_ responses, work rates, time to task failures, absolute and relative energy contributions of 100% of P_INC_, P_UPPERBOUND_, P_UPPERBOUND_´, I_HIGH_, ALP, and work rate performed at 5% above the ALP (ALP + 5%). Pairwise comparisons were made with a Bonferroni adjustment. A Greenhouse-Geiser correction was used where sphericity was violated. Effect size (ES) values for repeated-measures ANOVA were calculated as eta squared (η^2^) and considered *i)* minimum effect size if 0.04 < η^2^ ≤ 0.25; *ii)* moderate effect size if 0.25 < η^2^ ≤ 0.64; and *iii)* strong effect size if η^2^ > 0.64 (Ferguson 2009). A Pearson product-moment correlation was computed to evaluate the relationship between P_UPPERBOUND_, P_UPPERBOUND_´ and I_HIGH_ with the ALP. Additionally, standard error of estimation (SEE) and total error (TE) were used to examine the accuracy of estimations of the P_UPPERBOUND_, P_UPPERBOUND_´, I_HIGH_ and ALP (Lohman 1981; Hopkins 2000). Bland–Altman plots were used to assess the limits of agreement (LoA) between the P_UPPERBOUND_, P_UPPERBOUND_´, I_HIGH_ and ALP, and one-sample t-tests were used to determine whether the average difference between the values (i.e., Bias) was significantly different from zero (Bland and Altman 1986; Atkinson and Nevill 1998). Significance was set at *p* < 0.05. Results were reported as mean ± SD. According to the results of the post-hoc power analysis, based on a sample size of 15, alpha level of 0.05 and effect size of 0.84, the study had a sufficient statistical power (> 0.80) to detect meaningful differences (e.g., differences in work rates).

## Results

The highest 15-s $\dot{\text{V}}$O_2_ average obtained from the 100% of P_INC_ exercise (303 ± 48 W) was 54 ± 6 mL·min^−1^·kg^−1^, and there was no significant difference in $\dot{\text{V}}$O_2_ responses of 100% of P_INC_ and ALP (*p* > 0.05). However, $\dot{\text{V}}$O_2_ response to ALP + 5% exercise was significantly decreased (*p* < 0.01; ES: 0.63). The mean values for work rates, $\dot{\text{V}}$O_2_ responses, and time to task failures obtained from the P_UPPERBOUND_, P_UPPERBOUND_´, I_HIGH_, ALP, and ALP + 5% are represented in Table 1.

**Table 1:** Work rates, $\dot{\text{V}}$O_2_ responses, and time to task failures obtained from the P_UPPERBOUND_, P_UPPERBOUND_´, I_HIGH_, ALP, and ALP + 5% exercise

Variables	Work rate (W)	$\dot{\text{V}}$O_2_ (mL·min^−1^·kg^−1^)	TTF (s)
P_UPPERBOUND_	379 ± 61^bd^	−	123 ± 19^ac^
P_UPPERBOUND_´	384 ± 61^bd^	−	118 ± 19^ac^
I_HIGH_	339 ± 53	55 ± 7	211 ± 72
ALP	383 ± 58^bd^	53 ± 6	129 ± 12^ad^
ALP + 5%	401 ± 60^b^	50 ± 5^b^	104 ± 11^b^

P_UPPERBOUND_: the upper boundary of the severe exercise domain as the intersection of the relationship of the time to achieve $\dot{\text{V}}$O_2max_ plotted against time to task failure and the line of identity as proposed by Hill et al. (2002); P_UPPERBOUND_´: the upper boundary of the severe exercise domain as the intersection of the hyperbolic relationships between the time to achieve $\dot{\text{V}}$O_2max_ vs. power output and time to task failure vs. power output as suggested by Hill and Ferguson (1999), Hill et al., (2002) and Hill et al., (2024); I_HIGH_: the upper boundary of the severe exercise domain as the highest exercise intensity at which precalculated $\dot{\text{V}}$O_2max_ demand can be achieved based on the method proposed by Caputo and Denadai (2008); ALP: the highest power output at which the predominant energy contribution is derived from the aerobic energy system; ALP + 5%: constant work rate exercise performed 5% above the ALP; and TTF: time to task failure

^a^ Significantly different from the I_HIGH_, *p *< 0.01

^b^ Significantly different from the I_HIGH_, *p* < 0.001

^c^ Significantly different from the ALP + 5%, *p* < 0.01

^d^ Significantly different from the ALP + 5%, *p* < 0.001

**Fig. 5:**
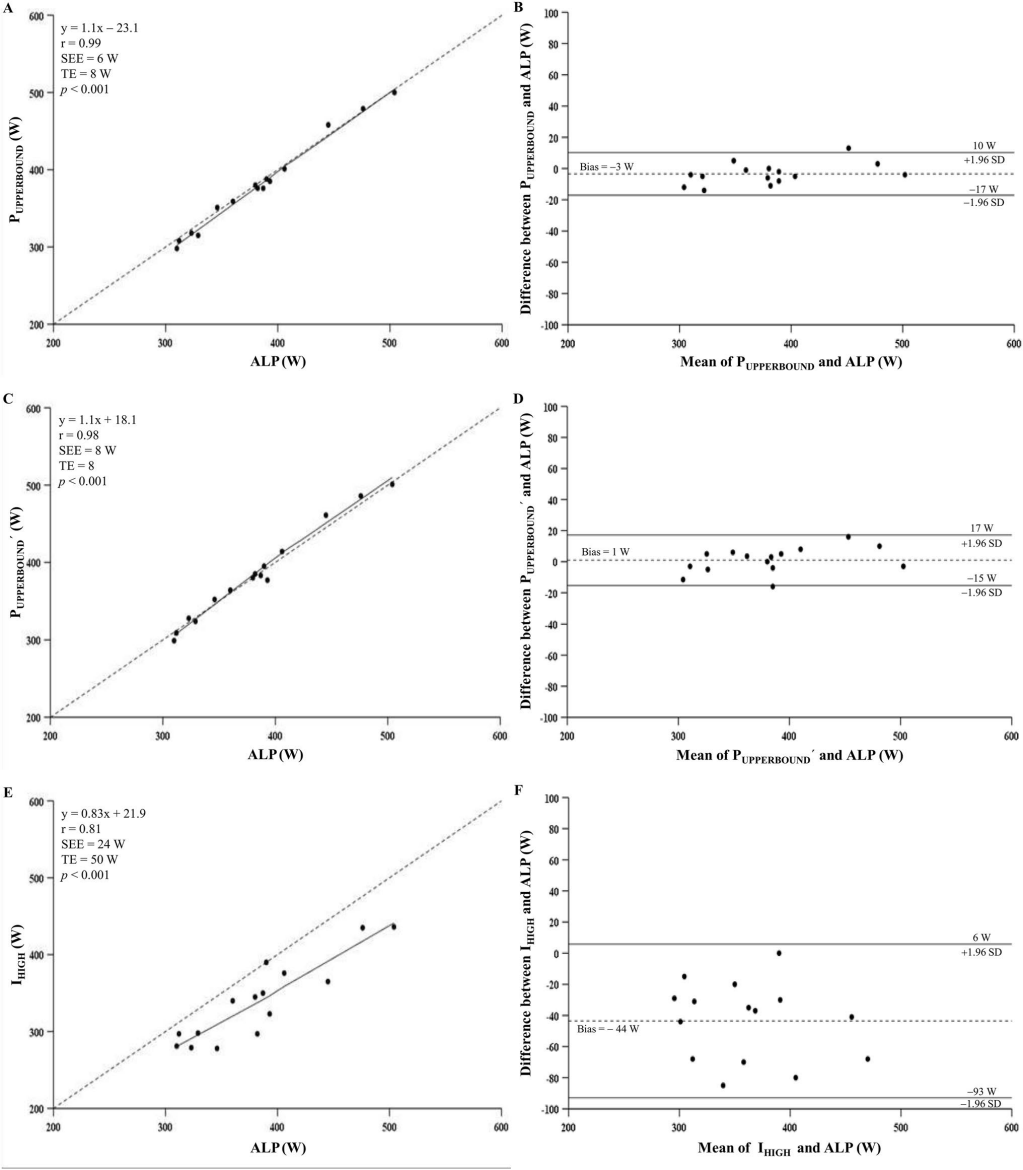
Correlation (**A**, **C** and **E**) and Bland–Altman analyses (**B**, **D** and** F**) between individual measures of the ALP and P_UPPERBOUND_, ALP and P_UPPERBOUND_´ and the ALP and I_HIGH_, respectively**.** In panels **A**, **C** and **E**, the solid lines represent the best-fit linear regression, and the dashed lines are the line of identity. In panels **B**, **D** and** F**, the solid lines represent the 95% limits of agreement, and the dashed lines are the mean difference between the two measures. P_UPPERBOUND_: the upper boundary of the severe exercise domain as the intersection of the relationship of the time to achieve $\dot{\text{V}}$O_2max_ plotted against time to task failure and the line of identity as proposed by Hill et al. (2002); P_UPPERBOUND_´: the upper boundary of the severe exercise domain as the intersection of the hyperbolic relationships between the time to achieve $\dot{\text{V}}$O_2max_ vs. power output and time to task failure vs. power output as suggested by Hill and Ferguson (1999), Hill et al., (2002) and Hill et al., (2024); I_HIGH_: the upper boundary of the severe exercise domain as the highest exercise intensity at which precalculated $\dot{\text{V}}$O_2max_ demand can be achieved based on the method proposed by Caputo and Denadai (2008); ALP: the highest power output at which the predominant energy contribution is derived from the aerobic energy system; and SEE: standard error of estimate. Data based on group means

The results and comparison of the markers showed that the P_UPPERBOUND_, P_UPPERBOUND_´ and ALP were similar in terms of both work rate and time to task failure values (*p* > 0.05), while the I_HIGH_ was significantly underestimated P_UPPERBOUND_, P_UPPERBOUND_´ and ALP (*p* < 0.001; ES: 0.84). Note that the difference was not significant between P_UPPERBOUND_ and P_UPPERBOUND_´, as expected (*p* > 0.05). Correlations, Bias, and LoA between the P_UPPERBOUND_, P_UPPERBOUND_´, I_HIGH_, and ALP are displayed in Fig. 5. According to the results, there were close agreements between ALP and P_UPPERBOUND_ (r: 0.99; Bias: − 3 W; SEE: 6 W; TE: 8 W; LoA: − 17 W to 10 W), and ALP and P_UPPERBOUND_´ (r: 0.98; Bias: 1 W; SEE: 8 W; TE: 8 W; LoA: − 15 W to 17 W).

**Fig. 6:**
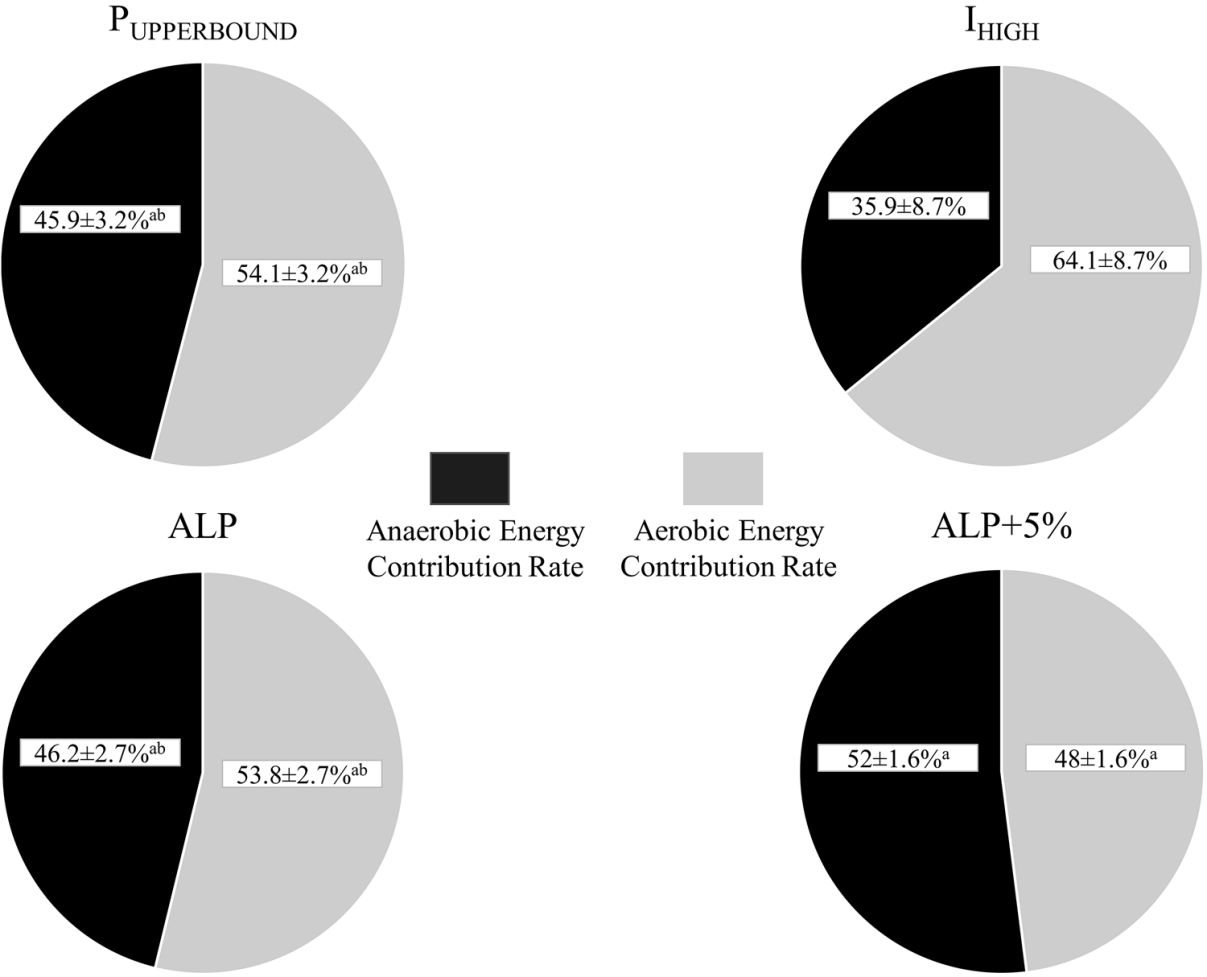
Relative contribution of the aerobic and anaerobic energy system in overall energy expenditure belonging to the P_UPPERBOUND_, I_HIGH_, ALP, and ALP + 5% exercise. P_UPPERBOUND_: the upper boundary of the severe exercise domain based on the method proposed by Hill et al. (2002); I_HIGH_: the upper boundary of the severe exercise domain based on the method proposed by Caputo and Denadai (2008); ALP: the highest power output at which the predominant energy contribution is derived from the aerobic energy system; and ALP + 5%: constant work rate exercise performed 5% above the ALP. Note that calculated contribution rates of energy systems were quite similar between P_UPPERBOUND_ and P_UPPERBOUND_´ (ES: 0.69 with < 0.9% differences between contribution rates) and not significantly different (*p* > 0.05). Data based on group means. ^a ^Significantly different from the I_HIGH_, *p* < 0.001. ^b ^Significantly different from the ALP + 5%, *p* < 0.01

As expected, there was a greater absolute aerobic energy contribution at the I_HIGH_ when compared to the P_UPPERBOUND_ (*p* = 0.017), P_UPPERBOUND_´ (*p* = 0.009) or ALP (*p* = 0.006) (Table 2). However, there were similar absolute aerobic energy contributions for the ALP, P_UPPERBOUND_ and P_UPPERBOUND_´ (*p* = 0.99). Otherwise, absolute contribution of aerobic energy system decreased significantly (Table 2), and therefore, relative anaerobic contribution became a predominant energy source (from 46 to 52%; *p* = 0.003; ES: 0.69) once the ALP was exceeded by 5%, i.e., during ALP + 5% exercise (Fig. 6).

**Table 2:** Parameters related to the absolute energy contribution of each energy pathway derived from the P_UPPERBOUND_, P_UPPERBOUND_´, I_HIGH_, ALP, and ALP + 5% exercise

Variables	P_UPPERBOUND_	P_UPPERBOUND_´	I_HIGH_	ALP	ALP + 5%
W_AER_ (kJ)	125 ± 18^ae^	120 ± 14^be^	212 ± 87	121 ± 17^be^	95 ± 16^c^
W_AER_ (mL·kg^−1^)	85 ± 11^ae^	81 ± 9^be^	144 ± 57	82 ± 10^be^	65 ± 10^c^
W_LA_ (kJ)	70 ± 12	69 ± 12	70 ± 11	70 ± 12	67 ± 14
W_LA_ (mL·kg^−1^)	48 ± 9	47 ± 9	48 ± 7	48 ± 9	46 ± 9
W_PC_ (kJ)	36 ± 10	36 ± 10	39 ± 8	34 ± 11	36 ± 14
W_PC_ (mL·kg^−1^)	24 ± 5	24 ± 6	26 ± 4	23 ± 6	24 ± 9
W_Total_ (kJ)	231 ± 26^ae^	225 ± 21^be^	320 ± 90	225 ± 26^bd^	198 ± 30^c^
W_Total_ (mL·kg^−1^)	157 ± 14^ae^	153 ± 13^be^	217 ± 58	153 ± 14^bd^	135 ± 19^c^

P_UPPERBOUND_: the upper boundary of the severe exercise domain as the intersection of the relationship of the time to achieve $\dot{\text{V}}$O_2max_ plotted against time to task failure and the line of identity as proposed by Hill et al. (2002); P_UPPERBOUND_´: the upper boundary of the severe exercise domain as the intersection of the hyperbolic relationships between the time to achieve $\dot{\text{V}}$O_2max_ vs. power output and time to task failure vs. power output as suggested by Hill and Ferguson (1999), Hill et al., (2002) and Hill et al., (2024); I_HIGH_: the upper boundary of the severe exercise domain as the highest exercise intensity at which precalculated $\dot{\text{V}}$O_2max_ demand can be achieved based on the method proposed by Caputo and Denadai (2008); ALP: the highest power output at which the predominant energy contribution is derived from the aerobic energy system; ALP + 5%: constant work rate exercise performed 5% above the ALP; W_AER_: the net energy contribution of the aerobic energy pathway; W_LA_: the net energy contribution of the lactic energy pathway; W_PC_: the net energy contribution of the phospholytic energy pathway; and W_Total_: the sum of the energy derived from W_AER_, W_LA_, and W_PC_. Note that the net energy contribution of aerobic, lactic and phospholytic energy pathway derived from the exercise performed at the P_UPPERBOUND_, P_UPPERBOUND_´, I_HIGH_, ALP, and ALP + 5% were significantly different among themselves

^a^ Significantly different from I_HIGH_, *p* < 0.05

^b^ Significantly different from I_HIGH_, *p* < 0.01

^c^ Significantly different from I_HIGH_, *p* < 0.001

^d^ Significantly different from ALP + 5%, *p* < 0.01

^e^ Significantly different from ALP + 5%, *p* < 0.001

## Discussion

**Fig. 7:**
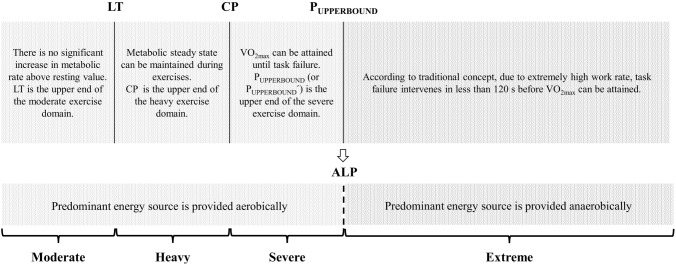
Conceptual advance of exercise intensity domain framework. The upper schematic illustration demonstrates exercise intensity domains and their boundaries. The lower schematic illustration presents conceptual advance of exercise intensity domains which are characterised by aerobic and anaerobic predominance. It should be considered that the ALP delineates predominantly aerobic intensity domain from anaerobic domain. Thus, it can be said that task failure intervenes in less than 120 s before aerobic energy contribution becomes main energy source within the extreme exercise domain. LT: lactate threshold, CP: critical power, P_UPPERBOUND_: the highest power output at which $\dot{\text{V}}$O_2max_ can be achieved, ALP: the highest power output at which predominant energy contribution is derived from the aerobic system, i.e., aerobic limit power

The aim of this study was to evaluate whether the highest exercise intensity at which predominant energy contribution is derived from the aerobic energy pathway (i.e., ALP) is a boundary that partitions the severe from extreme intensity exercise domain. Therefore, we compared the ALP to the P_UPPERBOUND_, P_UPPERBOUND_´ and I_HIGH_. To the best of our knowledge, the present study is the first to examine whether the ALP is associated with the upper boundary of the severe intensity exercise domain or not. Firstly, it should be noted that the ALP, as the upper end of the aerobic exercise zone, was sufficiently determined for each participant. While relative aerobic and anaerobic contribution rates of ALP intensity were 54% and 46% respectively, contribution rates were determined as 48% and 52%, once the ALP was exceeded by 5%, i.e., during ALP + 5% exercise. The principal novel findings demonstrated that the ALP was similar to the P_UPPERBOUND_ and P_UPPERBOUND_´ in terms of both work rates and time to task failures. Indeed, there were close agreements between the ALP and P_UPPERBOUND_, and ALP and P_UPPERBOUND_´. Otherwise, ALP, P_UPPERBOUND_ and P_UPPERBOUND_´ corresponded approximately 10–13% above the I_HIGH_, with a significant difference of 40–44 W between values (*p* < 0.001; ES: 0.84). In that case, it can be said that the ALP presents a novel approach to determine the upper boundary of the severe intensity exercise domain. Additionally, it offers a new perspective on the framework of exercise intensity domains, as it not only delineates the upper boundary of the severe intensity exercise domain but also indicates the upper end of the whole aerobic exercise zone including the moderate, heavy, and severe intensity exercise domains (Fig. 7).

It is known that $\dot{\text{V}}$O_2_ amplitude is constrained during an extreme intensity exercise, and anaerobic ATP resynthesis is relied upon to compensate for the energy deficit. In this case, anaerobic contribution preserves the energy contribution from oxidative metabolism. Indeed, the results of the present study indicated that once the ALP was exceeded by 5% (i.e., ALP + 5%), relative anaerobic energy measure became a major contributor (from 46 to 52%; *p* = 0.003). According to the literature, there could be a few reasons for the constriction of $\dot{\text{V}}$O_2_ response and increasing the anaerobic energy metabolism within the extreme intensity exercise domain. One plausible explanation for the decrease in final $\dot{\text{V}}$O_2_ amplitude observed during an extreme intensity exercise is the increase in relative contribution of type II fibres to muscular force production (Bottinelli and Reggiani 2000; He et al. 2000; Wilkerson et al. 2004). Type II fibres possess a lower oxidative capacity compared to type I fibres but have a higher glycolytic capability. Consequently, type II fibres may inherently exhibit a lower $\dot{\text{V}}$O_2_ amplitude and/or be compelled to fulfil a larger proportion of the energy requirements through the anaerobic energy pathways during exercise. Closely related with the increase in type II fibre recruitment, another plausible explanation is the fall in pH resulting from the accumulation of by-product in anaerobic metabolism (Poole et al. 1988; Vanhatalo et al. 2016; Bergstrom et al. 2017). This process inhibits oxidative phosphorylation and restricts the $\dot{\text{V}}$O_2_ rise during exercise (Conley et al. 2001; Jubrias et al. 2003). Therefore, anaerobic energy contribution in total energy expenditure increases during a typical extreme-intense exercise. The final explanation is the blood flow restriction (Krustrup et al. 2004). Depending on the increase in the work rate, the local motor unit recruitment increases at the constant work rate exercise performed within the extreme intensity exercise domain (Thomas et al. 2016; Zhang et al. 2021), thus the blood flow is restricted (Krustrup et al. 2004). That limits muscle oxygen delivery and obligates a greater anaerobic energy contribution (Wilkerson et al. 2004). These physiological factors may be the sign that there is a close relationship between the highest exercise intensity at which the predominant energy contribution is derived from the aerobic energy system and the highest exercise intensity at which $\dot{\text{V}}$O_2max_ can be achieved.

It should be noted that projected time to achieve $\dot{\text{V}}$O_2max_ was greater than realised time to task failure at which power output performed at ALP + 5% in present study (122 ± 18 vs. 104 ± 11 s; *p* > 0.001). This may also be a simplistic explanation to support that task failure occurred before $\dot{\text{V}}$O_2max_ can be achieved during exercise performed within the extreme intensity exercise domain (Ozkaya et al. 2023), and the ALP is identical to the highest power output at which $\dot{\text{V}}$O_2max_ can be attained, i.e., the upper boundary of the severe intensity exercise domain. This evidence also supports that the upper end of the aerobic exercise zone coincides with the upper boundary of the severe intensity exercise domain. Once the upper end is exceeded, neither a predominant (50%+) aerobic contribution nor a $\dot{\text{V}}$O_2max_ response is able to be obtained until task failure.

Hill et al. (2002) claimed that the greater the exercise intensity within the severe intensity exercise domain, the shorter the time to achieve $\dot{\text{V}}$O_2max_. Therefore, a unique work rate, at which $\dot{\text{V}}$O_2max_ would be attained, should be existed at the end of the domain. In this concept, the P_UPPERBOUND_ (or P_UPPERBOUND_´) provides the highest exercise intensity where $\dot{\text{V}}$O_2max_ can be achieved momentarily just before task failure. Indeed, based on our results, the P_UPPERBOUND_ and P_UPPERBOUND_´ have emerged as much more valid and plausible approaches compared to the I_HIGH_. However, P_UPPERBOUND_ and P_UPPERBOUND_´ include some mathematical approaches and presuppositions. For example, in these methods, the time to achieve $\dot{\text{V}}$O_2max_ values are originally calculated based on the 4.6 × τ value. Nevertheless, in some of our measurements, projected time to achieve $\dot{\text{V}}$O_2max_ values were higher than realised time to task failures belonging to the same exercise. For instance, for participant #4, projected time to achieve $\dot{\text{V}}$O_2max_ for 125% of P_INC_ was 136 s, while realised time to task failure associated with constant work rate exercise performed at 125% of P_INC_ was 117 s. This situation demonstrated that some individuals may have a slower (or faster) primary phase in $\dot{\text{V}}$O_2_ kinetics than expected. In the present study, the slope of the regression that provides the relationship between the time to achieve $\dot{\text{V}}$O_2max_ and time to task failure could not across the line of identity in some measurements. Therefore, we did exclude data obtained from two of our study applicants. In this respect, the inability to estimate the upper bound for the severe intensity exercise domain in some participants weakens the method.

Another limitation for the P_UPPERBOUND_ may be considered as R^2^ values of the relationships i.e., to engender the lower-than-expected R^2^ values (R^2^ < 0.85) of the relationship used to calculate the estimated time to task failure associated with the P_UPPERBOUND_. Indeed, for participant #3, R^2^ value of the slope of the regression line was 0.33, while the R^2^ values were found as 0.47 and 0.79 for participant #6 and participant #12, respectively. Indeed, Caputo and Denadai (2008) previously speculated that the relationship between the time to achieve $\dot{\text{V}}$O_2max_ value and time to task failure obtained from severe intensity exercise may be highly affected by aerobic training status of participants, and this may adversary affect R^2^ values of the relationships. Even though all these individual differences and mathematical presuppositions, the P_UPPERBOUND_ was highly correlated with the ALP, and both corresponded approximately 40–44 W above the I_HIGH_. Furthermore, the P_UPPERBOUND_ is a non-invasive method, while the ALP requires invasive approaches.

A similar investigation on behalf of the upper boundary of the severe intensity exercise domain was conducted by Caputo and Denadai (2008). The I_HIGH_ method bases on an interrogating $\dot{\text{V}}$O_2max_ demand. According to the authors, the $\dot{\text{V}}$O_2max_ demand implies a lower limit that must be strictly reached during exercise performed within the severe intensity exercise domain. However, calculated lower limit for $\dot{\text{V}}$O_2max_ has not always been attained during constant work rate exercise performed within the domain. For example, for participant #15, VO_2_ responses obtained from the four exercise tests (i.e., incremental exercise, 95%, 100% and 110% of P_INC_) were 4.04, 3.92, 3.99, and 3.89 L·min^−1^, respectively. Then, calculated *i)* VO_2_ average *ii)* standard deviation, and *iii)* typical error of measurement can be determined as 3.96 L·min^−1^, 0.07 L·min^−1^, 0.05 L·min^−1^, respectively. Finally, based on the I_HIGH_ method (qq. Eq. 3), VO_2max_ demand can be calculated as 3.91 L·min^−1^. However, calculated VO_2max_ could not be attained at 110% of P_INC_ exercise for participant #15. Moreover, it is also suspicious whether to attain precalculated $\dot{\text{V}}$O_2max_ value by a constant work rate exercise performed at 5% above the CP (i.e., CP + 5%, which provides the lowest exercise intensity within the severe intensity exercise domain) (Turnes et al. 2016b). Indeed, even in original study, authors reported that there was no expected time spent at $\dot{\text{V}}$O_2max_ during an interval exercise session performed at CP + 5% (Turnes et al. 2016b). Nevertheless, CP + 5% is a typical exercise intensity which provides the longest time spent at $\dot{\text{V}}$O_2max_. It can be said that the precalculated VO_2_ demand may be higher than the real VO_2_ response and could not represent to all severe intensity exercise tests, due to its relatively rigid methodology used in reliability evaluations.

Similar to the lactate threshold (i.e., LT) which delineates the transition from moderate to heavy exercise (Burnley and Jones 2007) or the maximal metabolic steady state (i.e., CP) which demarcates heavy from severe intensity domain (Jones et al. 2019), the identification of the upper boundary of the severe intensity exercise domain which denotes a transition from the severe to extreme, is of paramount importance for fitness assessment and training prescription (Hill et al. 2002; Hill and Stevens 2005). Some studies suggest that prescribing exercise intensity based on a certain fractional usage (%) of power output corresponding to $\dot{\text{V}}$O_2max_ may lead to highly heterogeneous physiological responses among individuals within the same exercise program (McLellan and Skinner 1985; Coyle et al. 1985; Meyer et al. 1999; Baldwin et al. 2000; Scharhag-Rosenberger et al. 2010; Lansley et al. 2011; Iannetta et al. 2020). Indeed, Inglis et al., (2024) recently mentioned that implementing exercise prescription based on the intensity domain framework can mitigate the confounding variability in the exercise intensity prescription, thereby reducing inter-individual differences in physiological and perceptual responses to training programs. Consequently, determining the power output that partitions the severe from extreme intensity exercise domain is imperative for normalising exercise intensity, and potentially minimising inter-individual variability in physiological and perceptual responses to exercise program.

## Conclusion

Firstly, P_UPPERBOUND_ (or P_UPPERBOUND_´) is a gold standard for the estimation of the upper boundary of the severe intensity exercise domain. It theoretically identifies the highest power output and the shortest exercise duration in which $\dot{\text{V}}$O_2max_ can be attained. Therefore, P_UPPERBOUND_ serves as a practical and physiological marker in exercise physiology, aiding both in the assessment of aerobic fitness and in the design of effective training regimens. Secondarily, ALP may provide a novel approach to understanding the significance of the upper boundary of the severe intensity exercise domain. Furthermore, it provides a new perspective to intensity domain framework since the ALP also indicates the upper end of the whole aerobic zone, i.e., moderate, heavy, and severe intensity exercise domains, and it may denote a transition from predominantly aerobic intensity zone to anaerobic intensity zone. Finally, coaches, athletes, or individuals should consider that the total anaerobic energy contribution increases and becomes predominant energy source within the total energy turnover compared to the aerobic rate during exercise performed just above that upper bound. In this case, the extreme intensity exercise might not provide a sufficient exercise induce to enhance aerobic power, and it is more appropriate to develop anaerobic performance related indices.

## Data Availability

Data generated and/or analysed during this study are available from the corresponding author upon reasonable request.

## Abbreviations

A_fc_: Amplitude of the fast component
ALP: Aerobic limit power
A_p_: Amplitude of the primary phase
A_sc_: Amplitude of the slow component
CP: Critical power
ES: Effect size
I_HIGH_: The upper boundary of the severe intensity exercise domain based on the method proposed by Caputo and Denadai (2008)
LoA: Limits of agreement
LT: Lactate threshold
P: Power output
P_INC_: Power output corresponding the highest $\dot{\text{V}}$O_2_ response measured during incremental tests
P_UPPERBOUND_: External power associated with the upper boundary of the severe intensity exercise domain as proposed by Hill et al. (2002)
P_UPPERBOUND′_: External power associated with the upper boundary of the severe intensity exercise domain as proposed by Hill and Ferguson (1999), Hill et al. (2002) and Hill et al., (2024)
SD: Standard deviation
SEE: Standard error of estimation
TD_fc_: Time delay during the fast component
TD_p_: Time delay during primary phase
TD_sc_: Time delay during slow component
TE: Total error
TTF: Time to task failure
$$\dot{\text{V}}$$O_2_: Pulmonary O_2_ uptake
$\dot{\text{V}}$O_2(t)_: $\dot{\text{V}}$O_2_ responses achievable at any time during exercise
$\dot{\text{V}}$O_2avg_: Average of the highest 15-s $\dot{\text{V}}$O_2_ values of incremental exercise and constant work rate exercise tests performed at 95%, 100% and 110% of P_INC_
$\dot{\text{V}}$O_2baseline_: $\dot{\text{V}}$O_2_ obtained from the end of the baseline cycling period
$\dot{\text{V}}$O_2max_: Maximal oxygen uptake
$\dot{\text{V}}$O_2resting_: $\dot{\text{V}}$O_2_ response during resting condition
$\dot{\text{V}}$O_2sd_: Standard deviation of the highest 15-s $\dot{\text{V}}{\text{O}_{2}}$values of incremental exercise and constant work rate exercise tests performed at 95%, 100% and 110% of P_INC_
W_AER_: Energy contribution of the aerobic energy pathway
W_ANE_: Energy contribution of the anaerobic energy pathway
W_LA_: Energy contribution of the lactic energy pathway
W_PC_: Energy contribution of phospholytic energy pathway
W_Total_: Total energy expenditure
η^2^: Eta squared
τ_fc_: Time constant of fast component
τ_p_: Time constant of primary phase
τ_sc_: Time constant of slow component
